# Antibiotic prophylaxis before tissue biopsy has no effect on culture results in presumed aseptic revision total hip arthroplasty

**DOI:** 10.5194/jbji-9-67-2024

**Published:** 2024-02-15

**Authors:** Jesse D. De Groot, Roy G. B. Brokelman, Bas L. Fransen, Tim U. Jiya, Dean F. M. Pakvis

**Affiliations:** Department of Orthopedic surgery, OCON Orthopedic Clinic, Geerdinksweg 144, 7555 DL Hengelo, the Netherlands

## Abstract

**Background**: Antibiotic prophylaxis (AP) is considered to be the gold standard for revision total hip arthroplasty (R-THA) due to the high incidence of prosthetic joint infection (PJI). To diagnose PJI, intraoperative tissue biopsies for culture are of particular importance. However, antibiotic interference could theoretically lead to less reliable culture results. Currently, there is no consensus on whether AP should be administered before or after tissue biopsy. In this study, we aimed to investigate the effect of AP timing on culture results and PJI rates in presumed aseptic R-THA. **Methods**: A retrospective single-center cohort study among 490 patients was performed; 61 patients received AP pre-incision, and 429 patients received AP post-biopsy. At least three intraoperative tissues were sampled for each patient and cultured for a minimum of 2 weeks. Minimum follow-up was 6 months. Epidemiological and clinical data (including culture results and incidence of PJI during follow-up) were gathered and analyzed. **Results**: Positive (4.9 % vs. 5.4 %, p=0.89 ) and contaminated culture results (23.0 % vs. 22.6 %, p=0.95 ) were not significantly different between pre-incisional and post-biopsy AP administration. Post-operative PJI incidence during follow-up was 1.6 % and 3.0 %, respectively. This difference was not statistically significant ( p=0.54 ). **Conclusion**: Pre-incisional AP administration does not yield fewer culture results compared to post-biopsy AP administration. Although statistically not significant, PJI during follow-up was almost twice as high when AP was withheld until after tissue biopsy. Other literature also supports the additional protective benefit of pre-incisional AP. Therefore, we believe pre-incisional AP administration is preferable for presumed aseptic R-THA.

## 1 Introduction

Prosthetic joint infection (PJI) is one of the most severe complications of revision total hip arthroplasty (R-THA) due to high mortality and revision rates, as well as a reduction in quality of life (Badarudeen et al., 2017; Wildeman et al., 2017). Additionally, septic indications for R-THA are almost twice as expensive as aseptic indications (Vanhegan et al., 2012; Abad and Haleem, 2017; Sousa et al., 2018). In the Netherlands, PJI accounted for 20.5 % of indications for R-THA between 2014 and 2021 (Dutch Arthroplasty Register Report, 2022). Due to the extensive impact of PJI, the risk of PJI following R-THA should be minimized as much as possible. Antibiotic prophylaxis (AP) has been an important part of PJI prevention (Tubb et al., 2020; Ricciardi et al., 2020; Gómez-Barrena et al., 2022). Several studies have indicated that AP timing influences the risk of developing surgical site infections, with the lowest risk being when AP is administered 15–60 min pre-incision (Van Kasteren et al., 2007; Weber et al., 2008; Nikolaus et al., 2016; De Jonge et al., 2017).

Accurate and timely diagnosis of a potential PJI is required for effective treatment. Intraoperative tissue cultures are therefore recommended for presumed aseptic R-THA, with two or more positive cultures of the same microorganism usually considered to be indicative of PJI (McNally et al., 2021). The incidence of unexpected positive intraoperative cultures (UPICs) in aseptic R-THA is 4 %–38 % (Renard et al., 2019; Schwarze et al., 2022), with Purudappa et al. (2019) reporting an average incidence of 10.5 %. However, AP might theoretically interfere with intraoperative culture results and therefore have a negative impact on the accurate diagnosis of PJI.

Within the current literature there is no consensus on the effect of AP on culture results in presumed aseptic R-THA. Some studies showed lower percentages of positive cultures when antibiotics were administered pre-incision, indicating a potentially higher occurrence of false-negative results (Malekzadeh et al., 2010; Wouthuyzen-Bakker et al., 2017a; Al-Mayahi et al., 2020). Other studies found no effect of AP on culture results (Tetreault et al., 2014; Bedenčič et al., 2016; Pérez-Prieto et al., 2016; Wouthuyzen-Bakker et al., 2017b). Due to discussions between orthopedic surgeons within our own medical center on the optimal timing of AP in presumed aseptic R-THA, we decided to perform a retrospective database study and analyze differences in culture results between patients receiving pre-incisional AP and patients receiving AP directly after tissue biopsy. Additionally, we investigated differences in post-operative PJI rates between these two study arms.

## 2 Methods

### 2.1 Study design and inclusion criteria

This single-center retrospective cohort study was performed at a high-volume orthopedic center in the Netherlands. Patients included underwent total or partial R-THA for presumed aseptic failure between January 2013 and December 2021. Pre-operative workup for infection consisted of clinical examination with radiological and laboratory studies, including infection markers such as C-reactive protein (CRP) and erythrocyte sedimentation rate (ESR) levels. In case of high clinical and/or radiological suspicion of PJI and/or elevated infection markers, synovial fluid aspiration or open tissue biopsy of the hip joint was performed for tissue culturing. R-THA was presumed to be aseptic in the case of negative culture results. Exclusion criteria were the following: (1) R-THA for septic indications, (2) antibiotic therapy less than 3 months before surgery, (3) fewer than three intraoperative cultures obtained, (4) follow-up less than 6 months post-surgery, and (5) missing essential epidemiological data.

### 2.2 Data collection

Data were collected retrospectively from the electronic patient files and the Dutch Arthroplasty Register (LROI). For all patients, basic epidemiologic data were collected: sex, age, body mass index (BMI), American Society of Anesthesiologists (ASA) score, total or partial R-THA, and the surgical indication. Further data collected were as follows: pre-operative CRP levels, pre-operative ESR levels, culture results of intraoperative tissue samples, microorganisms found in intraoperative tissue samples, and post-operative complications including PJI.

### 2.3 Antibiotics

Peri-operative AP was administered 15–60 min pre-incision or immediately after tissue biopsy. The timing of AP administration (pre-incision or post-biopsy) was based on the preference of the treating orthopedic surgeon. The standard AP agent used was cefazolin with weight-based dosing ( <80 kg, 1 g; <120 kg and ≥80 kg, 2 g; ≥120 kg, 3 g). In the case of cephalosporin allergy, vancomycin (20 mg kg^-1^) was administered instead of cefazolin according to national guidelines (Bauer et al., 2019). Antibiotics were continued for 48 h post-procedure (3 g cefazolin every 24 h if <80 kg or 6 g cefazolin every 24 h if ≥80 kg; the target serum concentration of vancomycin was 15–20 mg L^-1^).

### 2.4 Microbiology

Intraoperative tissue biopsy, depending on the component(s) revised, was performed at the (a) cup interface, (b) femur interface, (c) capsule, and (d) joint cavity. Tissue and fluid samples were stored separately. Each biopsy was performed with a new sterile surgical instrument. After collection, all samples were sent to the lab for assessment of quantity and quality. Microscopic analysis with gram staining was performed on the fluid samples. Tissue samples were cut into smaller portions with sterile scalpels and thereafter divided on several solid growth media non-selectively and selectively for gram-positive, gram-negative, and anaerobe bacteria. Enrichment cultures were also inoculated. Cultures were incubated for a minimum of 2 weeks, and bacterial growth was evaluated on set days. Matrix-assisted laser desorption/ionization time-of-flight mass spectrometry (MALDI-TOF MS) was used to identify bacteria (Alizadeh et al., 2021). Prior to inoculation, cultures were not sonicated. Furthermore, molecular techniques, such as polymerase chain reaction (PCR), were not utilized. Culture results were considered to be positive if a microorganism was found in two or more intraoperative tissue samples, contaminated if a microorganism of low virulence was found only within a single tissue sample, and negative when no microorganisms were found in any of the tissue or fluid samples. Additionally, if multiple colonies of a highly virulent microorganism (e.g., *Staphylococcus aureus*) were found in only one of the intraoperative tissue samples, the culture result was also considered to be positive.

### 2.5 Infection

For the diagnosis of PJI, the European Bone and Joint Infection Society's (EBJIS) definition of PJI was utilized (McNally et al., 2021). In the case of positive cultures, the presumed aseptic indication was considered to be septic, and antibiotic treatment was started accordingly in consultation with a physician–microbiologist specialized in orthopedic infections. PJI was considered to be a complication of the surgical procedure if it occurred within 6 months of surgery. Multidisciplinary team meetings (MDTMs) were held on a weekly basis for the diagnosis and management of potential or definitive PJIs.

### 2.6 Statistical analysis

To determine whether statistical differences existed between the two study arms, Chi-squared tests or Fisher's exact tests were used for categorical variables, and Mann–Whitney U tests were used for continuous variables. Additionally, Cochran–Mantel–Haenszel tests were used for the analysis of stratified categorical data. Statistical significance was defined as p<0.05 . All statistical analyses were performed using the R statistical software (R Foundation for Statistical Computing; Vienna, Austria; version 4.1.3).

## 3 Results

### 3.1 Demographics

**Figure 1:**
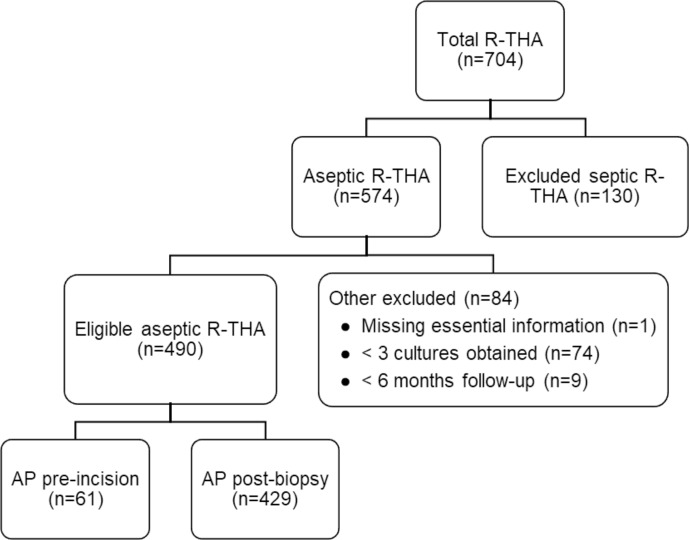
Flowchart of patient selection based on inclusion and exclusion criteria. Abbreviations: AP – antibiotic prophylaxis, R-THA – revision total hip arthroplasty.

A total of 704 patients underwent R-THA between January 2013 and December 2021, of which 574 were presumed to be aseptic. After application of other exclusion criteria, 490 patients were eligible for analysis. AP administration occurred pre-incision in 61 patients and post-biopsy in 429 patients. The exclusion rate was not statistically different between the two study arms (17.1 % vs. 18.0 %, p=0.87 ). An overview of our patient selection can be found in Fig. 1. The study arm with post-biopsy AP administration had significantly higher BMI (median [interquartile range]: 26.4 [14.4–57.0] vs. 28.0 [18.8–44.7], p=0.04 ) and fewer patients with an ASA score of I (2.1 % vs. 6.6 %, p=0.04 ). Although not significantly different, the number of patients with an ASA score of III or IV was lower in the study arm receiving pre-incisional AP (16.4 % vs. 26.2 %, p=0.09 ). Additionally, the number of obtained tissue samples was significantly lower in the study arm receiving pre-incisional AP (mean ± standard deviation: 4.9 ± 1.0 vs. 5.6 ± 1.6, p<0.01 ). No significant differences for age, sex, operational side (left or right), type of revision, and type of antibiotics were found between the two study arms. A full overview of all patient characteristics is displayed in Table 1. A total of 11 different experienced orthopedic surgeons were registered for all revisions.

**Table 1:** Overview of patient characteristics of the study arm receiving AP pre-incision versus the study arm receiving AP after tissue biopsy. Abbreviations: AP – antibiotic prophylaxis, ASA – American Society of Anesthesiologists, BMI – body mass index.

	AP pre-incision	AP post-biopsy	p -value
Patients, n	61	429	
Sex, n (%)			0.99
Male	20 (32.8)	143 (33.4)	
Female	41 (67.2)	286 (66.6)	
Age [range]	76 [37–89]	75 [37–97]	0.93
BMI [range]	28.0 [18.8–44.7]	26.4 [14.4–57.0]	0.04^a^
Operating side, n (%)			0.41
Left	34 (55.7)	215 (50.1)	
Right	27 (44.3)	214 (49.9)	
Type of revision, n (%)			0.62
Total	17 (27.9)	107 (25.0)	
Partial	44 (72.1)	322 (75.0)	
Type of antibiotics, n (%)			0.71
Cefazolin	61 (100)	428 (99.8)	
Vancomycin	0 (0)	1 (0.02)	
ASA score, n (%)			0.04^a^
I	4 (6.6)	9 (2.1)	0.04^a^
II	47 (77.0)	307 (71.7)	0.37
III or IV	10 (16.4)	113 (26.2)	0.09
Cultures obtained^b^ (SD)	4.9 (1.0)	5.6 (1.6)	$<0.01^{a}$

Special characters: ^a^ statistically significant, ^b^ average number of cultures taken per patient.

### 3.2 Culture result rates

The positive culture result rates of the pre-incisional and post-biopsy AP study arms were not significantly different (4.9 % vs. 5.4 %, p=0.89 ). The most predominant underlying microorganisms for positive culture results in both study arms were *Staphylococcus epidermis* (33.3 %, n=1 vs 52.2 %, n=12 ) and *Cutibacterium acnes* (66.7 %, n=2 vs. 21.7 %, n=5 ). Furthermore, contaminated culture results were also not significantly different between AP administration pre-incision and post-biopsy (23.0 % vs. 22.6 %, p=0.95 ). The most predominant underlying microorganisms for contaminated culture results in both study arms were *Cutibacterium acnes* (35.7 %, n=5 vs. 36.1 %, n=35 ) and *Staphylococcus epidermis* (28.6 %, n=4 vs. 24.4 %, n=24 ). Full overviews of all microorganisms found in positive and contaminated culture results are displayed in Tables 2 and 3, respectively. Additionally, stratification on BMI ( ≥30 and <30 ) and ASA score (I or II and III or IV) also did not lead to significant differences in positive ( p=0.83 and p=0.98 ) and contaminated culture results ( p=0.94 and p=0.93 ) between the two study arms . An overview of the stratified positive and contaminated culture results can be found in the Supplement (Tables S1 and S2).

**Table 2:** Overview of the total culture positive rate (at least two positive intraoperative cultures of a specific microorganism or multiple colonies of a single highly virulent microorganism in a single positive intraoperative culture) and per specific microorganism of the study arm receiving AP pre-incision versus the study arm receiving AP after tissue biopsy. Statistical differences are displayed accordingly. Abbreviations: AP – antibiotic prophylaxis.

Positive culture result rate:	AP pre-incision	AP post-biopsy	p -value
n patient(s) (%)	( n=61 )	( n=429 )	
Total positive culture results	3 (4.9)	23 (5.4)	0.89
*Staphylococcus epidermidis*	1 (33.3)	12 (52.2)	
*Cutibacterium acnes*	2 (66.7)	5 (21.7)	
*Staphylococcus aureus*	–	2 (8.7)	
*Staphylococcus lugdunensis*	–	2 (8.7)	
*Corynebacterium* spp.	–	2 (8.7)	
*Staphylococcus capitis*	–	1 (4.3)	
*Staphylococcus simulans*	–	1 (4.3)	
*Staphylococcus caprae*	–	1 (4.3)	
*Staphylococcus hominis*	–	1 (4.3)	
*Enterobacter cloacae*	–	1 (4.3)	
*Micrococcus luteus*	–	1 (4.3)	

**Table 3:** Overview of the total contamination rate (one positive intraoperative culture of a specific lowly virulent microorganism) and per specific microorganism of the study arm receiving AP pre-incision versus the study arm receiving AP after tissue biopsy. Statistical differences are displayed accordingly. Abbreviations – AP, antibiotic prophylaxis.

Contaminated culture result rate:	AP pre-incision	AP post-biopsy	p -value
n patient(s) (%)	( n=61 )	( n=429 )	
Total contaminated culture results	14 (23.0)	97 (22.6)	0.95
Total polymicrobial contaminated	2 (3.3)	23 (5.4)	0.76
*Cutibacterium acnes*	5 (35.7)	35 (36.1)	
*Staphylococcus epidermidis*	4 (28.6)	24 (24.7)	
*Staphylococcus capitis*	1 (7.1)	14 (14.4)	
*Staphylococcus caprae*	1 (7.1)	12 (12.4)	
*Dermacoccus* spp.	1 (7.1)	11 (11.3)	
*Veillonella* spp.	1 (7.1)	10 (10.3)	
*Staphylococcus pasteuri*	1 (7.1)	8 (8.2)	
*Staphylococcus hominis*	1 (7.1)	7 (7.2)	
*Kocuria rhizophila*	1 (7.1)	7 (7.2)	
*Staphylococcus warneri*	1 (7.1)	5 (5.2)	
Coagulase-negative staphylococci^*^	1 (7.1)	5 (5.2)	
Other microorganisms	5	36	

^*^ Not further specified in microbiological culture report.

### 3.3 Post-operative PJI rate

The incidence rates of PJI during follow-up of the pre-incisional and post-biopsy AP study arms were not significantly different (1.6 % vs. 3.0 %, p=0.54 ). Further analysis revealed that PJI during follow-up was also not significantly different between orthopedic surgeons ( p=0.85 ). Within the study arm receiving AP pre-incision, only one patient with a negative culture result and no patients with positive or contaminated results developed PJI during follow-up. As for the study arm receiving AP post-biopsy, three patients with positive culture results, two patients with contaminated culture results, and eight patients with negative culture results developed PJI during follow-up. All patients with positive culture results developed PJI during follow-up based on the microorganism found in the intraoperative cultures. Only one out of two patients with a contaminated culture result developed PJI during follow-up based on a microorganism previously considered to be contamination. A full overview of the incidence of PJI during follow-up per culture result is displayed in Table 4.

**Table 4:** Overview of post-operative PJI rate per culture result of the study arm receiving AP pre-incision versus the study arm receiving AP after tissue biopsy. Abbreviations: AP – antibiotic prophylaxis, PJI – prosthetic joint infection.

Post-operative PJI rate per culture	AP pre-incision	AP post-biopsy	Total
result: n/n patient(s) (%)	( n=61 )	( n=429 )	( n=490 )
Total	61/1 (1.6)	429/13 (3.0)	490/14 (2.9)
Positive	3/0 (0)	23/3 (13.0)	26/3 (11.5)
Contaminated	14/0 (0)	97/2 (2.2)	111/2 (1.8)
Negative	44/1 (2.3)	309/8 (2.6)	353/9 (2.5)

## 4 Discussion

In this retrospective cohort study of 490 patients, we evaluated the effect of AP timing on culture results and PJI rate in presumed aseptic R-THA. The aim of our research was to test the theory of whether AP administration pre-incision, and thus before tissue biopsy, would lead to less reliable culture results. In our current study, we found no significant differences in positive (4.9 % vs. 5.4 %, p=0.89 ) and contaminated culture results (23.0 % vs. 22.6 %, p=0.95 ) between patients receiving AP pre-incision or post-biopsy. Microorganisms found were also similar between the two study arms.

When comparing our findings with previous literature, there are some differences. Several studies reported a higher incidence of false negative culture results when antibiotics were administered before tissue cultures. For example, Malekzadeh et al. (2010) performed a retrospective case control study among 135 patients with culture-negative PJI matched with 135 patients with culture-positive PJI and found that antibiotic therapy less than 3 months before diagnosis was associated with an increased odds ratio (OR) of 4.7 for culture-negative PJI. Another retrospective case control study by Al-Mayahi et al. (2020) on 2740 episodes of orthopedic infections, of which 1167 patients (43 %) received pre-operative antibiotic therapy, found that pre-operative antibiotic exposure was associated with an increased OR of 2.8 for culture-negative results. It is important to note that antibiotics were administered therapeutically (range 1–14 d) in these studies instead of prophylactically like in ours. This potentially explains why different results were found. Interestingly, a systematic review by Wouthuyzen-Bakker et al. (2017a) on the effects of AP on intraoperative cultures among suspected and confirmed PJI cases found that culture yields were affected in 7 % of cases when AP was administered pre-incision. However, the clinical relevance of these findings was questioned by the authors when considering the fact that the risk of infection was even higher when AP was withheld. Their conclusion contained a recommendation for pre-operative AP, especially for patients with a low probability of infection who are undergoing revision arthroplasty (e.g., aseptic R-THA). Other studies specifically on AP in presumed septic revision arthroplasty found that pre-incisional AP did not significantly affect the UPIC rate (Tetrault et al., 2014; Bedenčič et al., 2016; Pérez-Prieto et al., 2016). Although these results are similar to our study, we believe our findings are more relevant as UPICs in aseptic revision have greater consequences for the post-operative treatment (e.g., long-term antibiotic treatment) than in septic revision. A retrospective cohort study by Wouthuyzen-Bakker et al. (2017b) on the effect of AP timing in presumed aseptic revision total knee arthroplasty (R-TKA) found no significant difference in positive culture results between pre-incisional and post-biopsy AP administration (26 % vs. 27 %), which is consistent with the findings reported in our study. Additionally, they found that, although not statistically significant, the post-operative infection rate in R-TKA was higher when AP was withheld until after tissue biopsy (6.4 % vs. 1.6 %). Our data also suggest a higher, yet again statistically insignificant, incidence of PJI during follow-up when AP is administered post-biopsy (3.0 % vs. 1.6 %, p=0.54 ). Other studies also suggest an additional protective benefit of pre-incisional AP against post-operative infection (Van Kasteren et al., 2007; Weber et al., 2008; Nikolaus et al., 2016; De Jonge et al., 2017). Based on previous mentioned findings, together with our data which suggest that pre-incisional AP does not lead to fewer culture results, we believe that withholding AP administration until after tissue biopsy in presumed aseptic R-THA exposes patients to an unnecessary higher risk of post-operative infection and that, therefore, pre-incisional AP is preferable. For septic revision procedures, pre-incisional AP may also potentially be considered. However, more research is needed before definitive recommendations can be made.

Our study has some limitations. First of all, this study was performed retrospectively. A prospective randomized controlled trial (RCT) would have increased the validity of our results. However, to acquire a similar number of patients for a prospective RCT would have required drastically more time and resources. Besides, our retrospective data give a good representation of the everyday practices of our and many other orthopedic clinics. Secondly, the timing of AP administration (pre-incision or post-biopsy) was by choice of the treating orthopedic surgeon. Therefore, our results could have been influenced by selection bias by the surgeons. However, all other peri-operative care was according to our R-THA protocol; therefore, we believe that the reliability of our results is not significantly impacted by this. Thirdly, a minimum of three intraoperative tissue samples was used as inclusion criteria in contrast to the five recommended by the EBJIS (McNally et al., 2021). Between 2013 and 2018, our R-THA protocol included a minimum of three intraoperative tissue samples based on recommendations from the Musculoskeletal Infection Society at that time (Parvizi et al., 2011). After the 2018 International Consensus Meeting on Musculoskeletal Infection, our infection work group decided to increase the minimum number of intraoperative tissue samples from three to five (Parvizi et al., 2018). To include more patients for our study, it was decided to set the minimum number of intraoperative tissue samples to three. Still, both study arms had an average of close to five samples per patient. Based on this, we suspect that this limitation did not significantly influence our findings. Fourthly, during our study period, only traditional culture methods were used for the detection of microorganisms. Advanced culturing techniques such as sonication and PCR could potentially have improved culture yields. Currently, sonication is increasingly used within our orthopedic clinic for the detection of microorganisms within intraoperative tissue samples of aseptic revision arthroplasty. Fifthly, study arm sample sizes were unequal. Although Chi-square and F statistics are sensitive to sample size, these tests remain quite robust with unequal sample sizes. Sixthly, BMI and ASA score were significantly different between the two study arms. Both variables influence the risk of post-surgical infection (Lenguerrand et al., 2018; Zhong et al., 2020). Stratification of the culture results based on these variables and subsequent statistical analysis did not yield significant differences between the two study arms. Unfortunately, due to low numbers, stratification and subsequent statistical analysis of the post-operative PJI rates were not possible. However, the higher BMI in the study arm receiving pre-incisional AP may have led to an underestimation of the protective benefit of pre-incisional AP against post-operative PJI. As for the difference in ASA score distribution, it is difficult to predict how this influences PJI rate as there was no significant difference between the number of patients with an ASA score ≥ III. Lastly, it is possible that some culture results were falsely classified as contamination. In fact, one of the PJIs during follow-up was caused by a microorganism which was previously presumed to be contamination. Some false classification is unavoidable, and we try to minimize this during our daily practice by regularly consulting physician–microbiologists specialized in orthopedic infections.

## 5 Conclusion

Analysis of a cohort of 490 patients who underwent presumed aseptic R-THA did not show less reliable culture results with AP administered pre-incision compared to post-biopsy. Additionally, although statistically insignificant, post-operative PJI rate was almost twice as high in patients who received AP post-biopsy. This study is the first of its kind, researching the effect of delayed AP administration in presumed aseptic R-THA on both culture results and PJI rates. Other literature also suggests an additional protective benefit against infection when AP is administered pre-incision. Therefore, we believe that, in the case of presumed aseptic R-THA, AP should not be withheld until after tissue biopsy and should be administered pre-incision to keep the risk of post-operative PJI as low as possible without affecting culture results. Future studies should further examine whether our findings are confirmed in a prospective randomized setting and if pre-incisional AP is also a valid option for septic revision procedures.

## Supplement

10.5194/jbji-9-67-2024-supplement The supplement related to this article is available online at: https://doi.org/10.5194/jbji-9-67-2024-supplement.

## Data Availability

Our hospital does not allow patient-related information to be stored in online repositories. However, an anonymized dataset is available upon request from the corresponding author.

## Code availability

The underlying software for the statistical calculations is available online at https://doi.org/10.6084/m9.figshare.25187648 (Bakker et al., 2024).
