# Supplementary material for: Antibiotic prophylaxis before tissue biopsy has no effect on culture results in presumed aseptic revision total hip arthroplasty
Source: J Bone Jt Infect. 2024 Feb 15;9(1):67–74. doi: 10.5194/jbji-9-67-2024 (PMC11004665; doi:10.5194/jbji-9-67-2024)
Supplement: The supplement related to this article is available online at: https://doi.org/10.5194/jbji-9-67-2024-supplement. [file jbji-9-67-supplement.pdf]

Supplement of J. Bone Joint Infect., 9, 67–74, 2024
https://doi.org/10.5194/jbji-9-67-2024-supplement
© Author(s) 2024. CC BY 4.0 License.

Supplement of

Antibiotic prophylaxis before tissue biopsy has no effect on culture results in presumed aseptic revision total hip arthroplasty

Jesse D. De Groot et al.

Correspondence to: Jesse D. De Groot (jd.de.groot@outlook.com)

The copyright of individual parts of the supplement might differ from the article licence.

SUPPLEMENTARY MATERIAL

Positive culture result rate: n patient(s) (%)	AP pre-incision (n=61)	AP post-biopsy (n=429)	p-value
BMI			0.83
<30	2 / 38 (5.3)	16 / 312 (5.1)	
≥30	1 / 20 (5.0)	7 / 94 (7.4)	
ASA			0.98
I/II	3 / 48 (6.3)	13 / 303 (4.3)	
III/IV	0 / 10 (0)	10 / 103 (9.7)	

Table S1: Overview of the total culture positive rate (at least two positive intraoperative cultures of a specific microorganism or multiple colonies of a single high virulent microorganism in a single positive intraoperative culture) stratified by BMI (≥30 or <30) and ASA (I/II or III/IV) of the study arm receiving AP pre-incision versus the study arm receiving AP after tissue biopsy. Cochran-Manzel-Haenszel tests were applied on the stratified data and statistical differences are displayed accordingly. Abbreviations: AP, antibiotic prophylaxis.

Contaminated culture result rate: n patient(s) (%)	AP pre-incision (n=61)	AP post-biopsy (n=429)	p-value
BMI			0.94
<30	13 / 27 (48.1)	75 / 254 (29.5)	
≥30	1 / 20 (5.0)	20 / 80 (25.0)	
ASA			0.93
I/II	11 / 40 (27.5)	72 / 244 (29.5)	
III/IV	3 / 7 (42.9)	24 / 89 (27.0)	

Table S2: Overview of the total contamination rate (one positive intraoperative culture of a single low virulent microorganism) stratified by BMI (≥30 or <30) and ASA (I/II or III/IV) of the study arm receiving AP pre-incision versus the study arm receiving AP after tissue biopsy. Cochran-Manzel-Haenszel tests were applied on the stratified data and statistical differences are displayed accordingly. Abbreviations: AP, antibiotic prophylaxis.
